# Genomic prediction of zinc-biofortification potential in rice gene bank accessions

**DOI:** 10.1007/s00122-022-04110-2

**Published:** 2022-05-26

**Authors:** Mbolatantely Rakotondramanana, Ryokei Tanaka, Juan Pariasca-Tanaka, James Stangoulis, Cécile Grenier, Matthias Wissuwa

**Affiliations:** 1 Rice Research Department, The National Center for Applied Research on Rural Development (FOFIFA), 101 Antananarivo, Madagascar; 2 Department of Agricultural and Environmental Biology, Graduate School of Agricultural and Life Sciences, The University of Tokyo, 1-1-1 Yayoi, Bunkyo, Tokyo, 113-8657 Japan; 3 Crop, Livestock and Environment Division, Japan International Research Center for Agricultural Sciences (JIRCAS), 1-1 Ohwashi, Tsukuba, Ibaraki 305-8686 Japan; 4 College of Science and Engineering, Flinders University, Bedford Park, SA 5042 Australia; 5 CIRAD, INRAE, Institut Agro, UMR AGAP Institut, Univ Montpellier, 34398 Montpellier, France

## Abstract

**Key message:**

A genomic prediction model successfully predicted grain Zn concentrations in 3000 gene bank accessions and this was verified experimentally with selected potential donors having high on-farm grain-Zn in Madagascar.

**Abstract:**

Increasing zinc (Zn) concentrations in edible parts of food crops, an approach termed Zn-biofortification, is a global breeding objective to alleviate micro-nutrient malnutrition. In particular, infants in countries like Madagascar are at risk of Zn deficiency because their dominant food source, rice, contains insufficient Zn. Biofortified rice varieties with increased grain Zn concentrations would offer a solution and our objective is to explore the genotypic variation present among rice gene bank accessions and to possibly identify underlying genetic factors through genomic prediction and genome-wide association studies (GWAS). A training set of 253 rice accessions was grown at two field sites in Madagascar to determine grain Zn concentrations and grain yield. A multi-locus GWAS analysis identified eight loci. Among these, QTN_11.3 had the largest effect and a rare allele increased grain Zn concentrations by 15%. A genomic prediction model was developed from the above training set to predict Zn concentrations of 3000 sequenced rice accessions. Predicted concentrations ranged from 17.1 to 40.2 ppm with a prediction accuracy of 0.51. An independent confirmation with 61 gene bank seed samples provided high correlations (*r* = 0.74) between measured and predicted values. Accessions from the *aus* sub-species had the highest predicted grain Zn concentrations and these were confirmed in additional field experiments, with one potential donor having more than twice the grain Zn compared to a local check variety. We conclude utilizing donors from the *aus* sub-species and employing genomic selection during the breeding process is the most promising approach to raise grain Zn concentrations in rice.

**Supplementary Information:**

The online version contains supplementary material available at 10.1007/s00122-022-04110-2.

## Introduction

Zinc (Zn) is an essential element for plants and humans alike, because Zn is a component of thousands of enzymes and a key regulator of gene expression and protein synthesis (Broadley et al. 2007; Galetti 2018). Zinc malnutrition is a global health problem that is particularly serious in infants where it impairs immune system function and delays infant development, causing stunting as the most visible symptom (Roohani et al. 2013; Galetti 2018). Alleviating human malnutrition for Zn has been included as one of the top priorities in the Sustainable Development Goals (SDG 2.2: End all forms of malnutrition). In 2003 the Consultative Group on International Agricultural Research (CGIAR) initiated a program to breed crops with higher concentrations of Zn, Fe and Pro-vitamin A carotenoids in the edible parts of a crop, an approach termed biofortification (Bouis and Saltzman 2017). Biofortification of crops represents an alternative to food fortification and while both approaches are important in alleviating malnutrition, it is believed that crop biofortification is a very efficient tool to reach rural communities that are largely food self-sufficient (Virk et al. 2021). Programs to develop Zn biofortified rice varieties have been successful in Asia (Swamy et al. 2016; Rao et al. 2020) and Latin America (Harvest Plus 2021) but concerted efforts to do so in Africa are still non-existing.

Madagascar remains a low-income country with a high level of malnutrition (The World Bank 2016; WFP 2010). In rural areas, 50% of children suffer from stunting and are underweight (Stewart et al. 2020), one of the highest rates in the world (UNICEF 2019). In the central highlands, the highest levels of stunting (60%) are found and recent surveys by JIRCAS and partners estimated that 80% of the population consume inadequate amounts of Zn (Shiratori et al. 2018). Rice is essential in Malagasy diets; it is eaten three times a day and represents 50 percent of the daily caloric intake with per capita consumption being above 120 kg annually. Rice, having such a pre-eminent position for food supply, is naturally a target for intervention. Consequently, Madagascar has the 3rd highest Biofortification Prioritization Index (BPI) for Zn in rice for Africa and the 13th highest globally (Harvest Plus 2021).

Zn concentrations in polished rice are typically too low to supply a sufficiently high proportion of the daily required intake of Zn (Bouis and Welch 2010), thus where rice is the main staple and households cannot afford to diversify their meal by adding mineral-rich fruits, vegetables and meat, Zn deficiency is prevalent (Harvest Plus 2021). To overcome this deficiency, grain Zn concentration in rice needs to be increased by 50% or more to significantly alleviate Zn malnutrition (Bouis and Welch 2010). Developing rice varieties with increased grain Zn concentrations therefore remains an important global objective (Rao et al. 2020) that offers a low-cost and long-lasting solution to the persisting problem of Zn malnutrition (Bouis et al. 2011).

Rice grain Zn concentrations are strongly affected by factors such as genotype and environment, with soil properties being the main source of environmental variation. For a given genotype, grain Zn concentrations may vary by a factor 2–3 depending on soil type and related Zn bio-availability for plant uptake (Wissuwa et al. 2008; Goloran et al. 2019; Rao et al. 2020). Low Zn bio-availability in paddy soils is commonly associated with alkalinity (high soil pH and excess bicarbonate) and very low soil redox potentials (Johnson-Beebout et al. 2016). Both factors trigger the formation of Zn-complexes with soil constituents and in consequence the soluble Zn fraction that is removed by the plant will be replenished too slowly to assure high Zn uptake rates (Broadley et al. 2007). The effect of a decreasing soil redox potential after flooding tends to cause Zn bio-availability to be lowest toward the end of the cropping season and thus reduces Zn uptake during the reproductive phase when Zn taken up may be directly transported to reproductive organs. For this reason, basal Zn fertilizer application has often very limited effects on increasing grain Zn concentrations (Johnson-Beebout et al. 2016) and it would explain the observation that grain Zn concentrations tend to be lower during the rainy season compared to the dry season (Goloran et al. 2019).

The genotypic variation in grain Zn concentrations is similar in magnitude to the environmental variation with 2–3 fold differences having been detected repeatedly (Norton et al. 2014; Swamy et al. 2018; Zhang et al. 2018). Since grain Zn concentrations are influenced at many levels, starting with Zn uptake by the root, followed by transport and reallocation of Zn within the plant, to Zn loading into the grain (Swamy et al. 2016), it is likely the genotypic differences at each of these levels exist. Which of these factors contribute most to genotypic differences in grain Zn concentrations remains uncertain. Some high-Zn genotypes appear to rely mostly on Zn remobilization, whereas others maintain high Zn uptake rates during grain filling (Johnson-Beebout et al. 2016). At the same time, increased root uptake does not necessarily result in enhanced Zn accumulation in rice grains, suggesting Zn loading into the endosperm to be the main limiting step for which genotypic differences fortunately exist (Jiang et al. 2008).

Nicotianamine (NA) is a ubiquitous chelator of metal cations, such as Fe^2+^ and Zn^2+^. Biosynthetic precursor of phytosiderophore secretion from roots, NA is responsible for Fe internal metal transport and maintenance of metal homeostasis. In rice, three NA synthase genes were identified (*OsNAS1*, *OsNAS2* and *OsNAS3*) that have been largely studied to demonstrate their role in increased bioavailable Fe levels in rice grains (Higuchi et al. 2001; Inoue et al. 2003). Through transgenic approaches overexpressing the *OsNAS* gene, it has been possible to significantly increase both grain Fe and Zn concentration, indicating Zn transport processes to be of additional importance (Johnson et al. 2011).

The genetic control of grain Zn concentration in rice has been widely studied, using bi-parental mapping populations (reviewed in Swamy et al. 2016), diversity panels (Norton et al. 2014; Zhang et al. 2018) or double-haploid (DH) derived biparental populations (Swamy et al. 2018). Consistently, these studies reported a large number of genetic regions controlling Zn concentration, each with relatively minor effects. This may be expected given that grain Zn concentrations are likely the result of multiple interacting physiological processes. For Zn uptake alone, it has been shown that at least two distinct processes, root proliferation and rhizosphere Zn mobilization, are causative of genotypic differences in plant Zn uptake (Mori et al. 2016). While none of the identified loci appear to be currently used in marker-assisted breeding, loci on chromosomes 7, 11 and 12 have been identified consistently (Swamy et al. 2016). Of these, the QTL on chromosome 7 co-localizes with *OsNAS3* and may therefore be of particular interest (Cu et al. 2021).

The complex nature of a trait like grain Zn concentration, which depends on multiple physiological mechanisms, each potentially controlled by multiple underlying genes, may necessitate a genome-wide rather than a single marker selection approach. Genomic Prediction (GP; Meuwissen et al. 2001) for mineral content has already proven efficient in maize and wheat where the predictive ability (PA) for grain Zn content was between 0.43 and 0.73 in maize (Mageto et al. 2020) and between 0.33 and 0.69 for wheat (Velu et al. 2016) depending on the population and environment chosen. A similar PA of 0.51 was achieved for grain Zn concentration improvements in a rice synthetic population managed through recurrent selection when multi-site data were considered for the calibration model (Baertschi et al. 2021).

Given that environmental factors strongly affect grain Zn concentrations, it is of interest to determine to what extent GP can be employed in target environments that are less homogenous compared to the well-managed trials conducted on research stations. While the polygenic nature of grain Zn may favor a GP approach, it is possible that main effect single loci are more stable across environments and therefore possess greater predictive power in less controlled environments. We have grown a set of 253 rice gene bank accessions sampled from the 3 K genome project (Mansueto et al. 2017) in two farmer’s fields in Madagascar and determined the variation for grain Zn concentrations and grain yield. Using this dataset, the objectives of this study were to:

(i) Conduct genome-wide association studies (GWAS) in an attempt to detect alleles associated with high grain Zn concentrations,
(ii) Develop a GP model for grain Zn concentrations based on above training set and employ this model to predict grain Zn concentrations among the 3000 sequenced rice accessions available at the IRRI gene bank,
(iii) Identify potential donors with high grain Zn concentrations and confirm their suitability through confirmatory experiments.

## Materials and methods

### Field phenotyping

Field experiments were conducted at two sites in the central highlands of Madagascar, Anjiro (elevation 950 m, 18°54′01.7 ′′S 47°58′12.4 ′′E) and Ankazomiriotra (1150 m, 19°40′07.9 ′′S 46°33′53.9 ′′E). The experiments were carried out in farmers' fields under flooded lowland conditions during the 2017–18 rainy season with sowing in November, transplanting in late November to December and harvests in April to May. Following the typical farmer’s practice in the region, chemical fertilizer was not applied and neither did fields receive organic manure. At each site, 523 accessions selected from the set of 3 K sequenced accessions available at IRRI were grown with two replications in a randomized complete block design. Several sub-sets selected to represent extreme variation for grain yield, maturity or plant height existed within these 523 accessions, (Tanaka et al. 2021) and only those considered adapted to our field sites were used in the present study (see below).

At both sites, accessions were transplanted in 2-row micro-plots of 2 m length with spacing of 20 cm between and within rows (22 single plant hills per plot). Heading date (HD) was recorded at 50% heading for each accession. During harvest, five representative plants per plot were cut, panicles were separated from straw, placed in paper bags to avoid contamination by soil or dust, and taken to the laboratory where they were air-dried for a week before total panicle dry weight was determined. Grain yield (GY) was estimated from the panicle weight of these five plants, assuming a realized density of 22 hills per m^2^ and expressed in kg per ha. Straw weight (SWT) was determined on the same five harvested plants, first as fresh weight which was then adjusted for moisture content after oven-drying a sub-sample for 48 h at 70 °C.

### Grain processing and grain Zn analysis

Grain Zn concentrations were determined for a subset of 253 accessions from the 523 grown at field sites. All accessions considered poorly adapted to experimental sites were omitted, which included accessions with very early or late maturity and all accessions with low GY or that had lodged and had been contaminated by soil. A focus on well-adapted accessions was meant to prevent the potential confounding effect of high grain Zn being the result of poor grain yield and very low harvest index. A random sub-sample of the harvested grain from ten panicles per plot was dehulled to obtain brown rice and these whole grain samples were sent to Flinders University, Australia for further analysis.

For inductively coupled plasma mass spectrometry (ICP-MS) analysis, 0.3 g of whole rice seed, which had been oven-dried at 80 °C for 4 h to remove remaining moisture, was acid-digested in a closed tube as described in Wheal et al. (2011). Elemental concentrations of samples were measured using ICP-MS (8900; Agilent, Santa Clara, CA) according to the method of Palmer et al. (2014). The grain Zn concentration is given in μg g^−1^ on a dry weight basis. In each of 10 digestion batches, a blank and a certified reference material (CRM; NIST 1568b rice flour) were added for quality assurance. Samples with Al present at > 5 μg g^−1^ were considered to have unacceptable levels of purported soil contamination (Yasmin et al. 2014), thus they were eliminated from the dataset.

### Statistical analysis for phenotypic values

Given the experimental design, the following linear model was fitted for each trait:

$$y_{ijk} = \mu + g_{i} + s_{ij} + \alpha_{j} + \beta_{jk} + e_{ijk}$$

where $y_{ijk}$ is the phenotypic value (*i.e.*, observed Zn concentration) of *i*-th genotype evaluated at the *k*-th block in *j*-th site, $\mu$ is an intercept, $g_{i}$ is the genotypic value of *i*-th genotype, $s_{ij}$ is the interaction effect between the *i*-th genotype and the *j*-th site, $\alpha_{j}$ is the effect of *j*-th site, $\beta_{jk}$ is the effect of *k*-th block in the *j*-th site, and $e_{ijk}$ is the residual. The interaction effects $s_{ij}$ and the block effects $\beta_{jk}$ were modeled as random effects, and the other model terms were modeled as fixed effects. This model was implemented in *lmer* function in the lme4 package. To test the statistical significance between two sites, site effect ($\alpha_{j}$) for each trait was tested based on the type-III analysis of variance with Satterthwaite's method using the *anova* function in the lmerTest package (Kuznetsova et al. 2017). The estimated values (best linear unbiased estimates; BLUEs) of $g_{i}$ were used in the subsequent association mapping and the genomic prediction analyses.

Heritability was calculated based on the same linear model but treating the genotypic values $g_{i}$ as random effects. Using the estimated variance components of the genotypic values ($s_{ij}$), genotype-by-site interaction effects ($\sigma_{s}^{2}$) and residuals ($\sigma_{e}^{2}$), a broad-sense heritability ($H^{2}$) was calculated as follows (Holland et al. 2003);

$$H^{2} = \frac{{\sigma_{g}^{2} }}{{\sigma_{g}^{2} + \frac{{\sigma_{s}^{2} }}{{n_{{{\text{site}}}} }} + \frac{{\sigma_{e}^{2} }}{{n_{{{\text{block}}}} \times n_{{{\text{site}}}} }}}}$$

where $n_{{{\text{block}}}}$ is the number of blocks per site, $n_{{{\text{site}}}}$ is the number of sites (*i.e.*, $n_{{{\text{block}}}} = n_{{{\text{site}}}} = 2$ given our experimental design).

Phenotypic correlation among sites and traits was calculated based on the Pearson’ correlation after averaging the observed phenotypic values over the two blocks for each accession (if an accession did not have an observed value in one of the two blocks, the available phenotypic value was used instead of the average).

### Genomic data and Genome-Wide Association (GWA) analysis

The 404 K core SNPs dataset was downloaded from the IRRI SNP-Seek website (https://snp-seek.irri.org/_download.zul). SNP having more than 5% missing data or a minor allele frequency below 2.5% were removed, retaining 186,229 SNPs for 3,024 accessions. Remaining missing states were imputed using Beagle v.4.1 (21Jan17.6 cc; Browning and Browning 2016).

Without further filtering, GWA analysis was performed on the 253 accessions with the BLUE values for grain Zn concentrations and the 186 k genotype matrix using the multi-locus random-SNP-effect mixed linear model (mrMLM) software package, which includes the mrMLM, FASTmrMLM, FASTmrEMMA, pkWmEB, pLARmEB, ISIS EM-BLASSO methods (https://cran.r-project.org/web/packages/mrMLM/index.html). A kinship matrix was calculated by mrMLM by default using the method of Kang et al. (2008) and default values were used for the parameters in all methods. To account for additional population structure a set of principal components (PC) was calculated using TASSEL (v5.2.75). PCs explaining more than 5% of the variation were included in the GWA analysis by indicating the type of population structure (PopStrType) = “PCA.” An output of Quantitative Trait Nucleotides (QTN) exceeding a threshold LOD value > 3 at each of the six multi-locus models was generated as the last step of the analysis and visualized in a combined Manhattan plot. QTN exceeding this LOD threshold in at least three of the six models were considered significant and evaluated further.

The allele effect at each locus was determined by calculating the average phenotypic values of all accessions carrying either allele and a box-plot graph was generated using an in-house R script. A graphical representation of subset of SNPs surrounding the significant QTNs was generated by the Haploview 4.2 software (Barret et al. 2005). Linkage disequilibrium (LD) blocks were then identified and manually delignated based on the recombination rate, which is displayed using the standard color scheme: *D*’/LOD (wherein red color reveals linkage disequilibrium between two genetic markers, *D*' = 1 and LOD > 2).

### Genomic prediction

Genomic prediction was performed with the GBLUP model (Bernardo 1994) using the rrBLUP package (Endelman 2011):

$${\mathbf{g}} = 1{\upmu } + {\mathbf{Zu}} + {\mathbf{e}}, \;\;\; {\mathbf{u}} \sim {\text{MVN}}\left( {0,{ }{\mathbf{G}}{\upsigma }_{u}^{2} } \right),\;\; {\mathbf{e}} \sim {\text{MVN}}\left( {0,{ }{\mathbf{I}}{\upsigma }_{e}^{2} } \right)$$

where $\mathbf{g}$ is the Zn BLUE values, $1$ is a vector of ones, $\upmu$ is the grand mean, $\mathbf{Z}$ is the design matrix, $\mathbf{u}$ is the vector of genotypic values, $\mathbf{e}$ is the vector of residuals, $0$ is a vector of zeros, $\mathbf{G}$ is a genomic relationship matrix, ${\upsigma }_{u}^{2}$ is genetic variance, $\mathbf{I}$ is an identify matrix, and $\sigma_{e}^{2}$ is the residual variance. The genotypic values and residuals are assumed to follow a multivariate normal distribution (MVN). The genomic relationship matrix was calculated by using the A.mat function in rrBLUP package, as described in Tanaka et al. (2021).

By using the above GBLUP model, Zn concentrations of the all 3 K accessions were predicted with three slightly different training sets: (1) all phenotyped accessions (*n* = 253), (2) all phenotyped accessions excluding IRIS_313_9368, which had extremely high Zn concentrations and may therefore be highly influential (*n* = 252) and (3) excluding the six members of the *aus* subspecies (*n* = 247). In addition, tenfold cross-validation was repeated 10 times to evaluate the prediction accuracy within the phenotyped accessions. Prediction accuracy was evaluated by taking correlation between the BLUE values ($g$) and the predicted genotypic values from the GBLUP model ($u$).

### Confirmatory experiments

Two subsequent experiments were conducted to confirm results obtained with the training set. To independently confirm the reliability of the GP model, 61 additional accessions from the 3 K set available at IRRI were selected for the determination of grain Zn concentrations. These accessions were selected based on their predicted grain Zn concentrations being either high (*n* = 19), intermediate (*n* = 24) or low (*n* = 18). Accessions had not been grown in Madagascar but seed produced at IRRI and distributed to JIRCAS was used directly for the determination of grain Zn concentrations. After acid-digestion of 0.25 g dehulled seed, elemental concentrations in samples were measured using ICP Emissions Spectrometer ICPE- 9000 (Shimadzu, Kyoto, Japan).

A further experiment was conducted to investigate whether the high grain Zn concentrations identified in potential high-Zn donor accessions were repeatable and stable across several field sites in Madagascar. Three high-Zn accessions of the training set (IRIS313-9368, IRIS313-10,114 and CX158) were grown in a multi-location trial together with local (X265) and international (IR64) check varieties. The experiments were conducted in Anjiro, Ankazo and Behenji (elevation 1428 m, 19°14′44.92′′S 47°28′45.38′′E) villages in the central highlands of Madagascar during the 2018–19 rainy season, using five farmer’s fields with two fertilizer treatments (zero input versus fertilization with NPK) and three replications. Plot sizes were 2 m^2^. Grain samples were processed as for the training set and sent to Flinders University for the determination of elemental concentrations as outlined above.

## Results

### Phenotypic variation

**Fig. 1:**
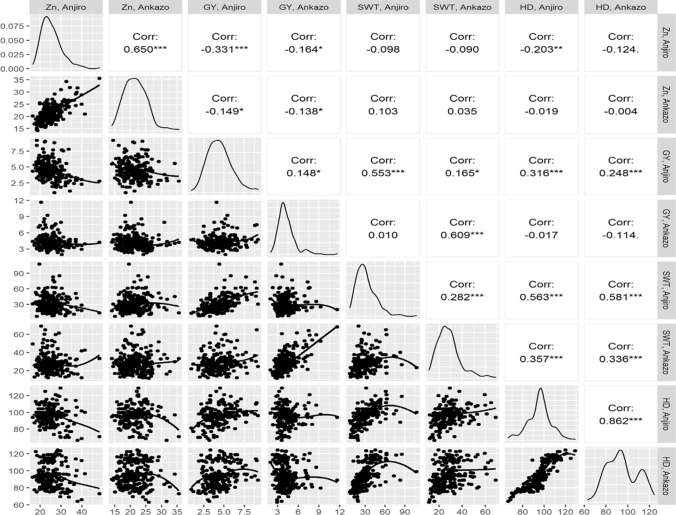
Repartition and correlation of zinc concentration (Zn), grain yield (GY), shoot weight (SWT) and heading date (HD) at sites Ankazomiriotra (AZ) and Anjiro (AJ)

The average grain Zn concentrations (Zn) for the 253 tested accessions ranged from 16.6 to 48.4 µg g^−1^ at the Anjiro site and from 14.2 to 35.6 µg g^−1^ at the Ankazo site (Fig. 1) and highest values at both sites were detected in accession IRIS_313_9368. In addition to having wider variation, the Anjiro site average of 25.4 µg g^−1^ was significantly higher (*p* < 0.01) compared to Ankazo with 21.6 µg g^−1^. Despite these differences, grain Zn concentrations at both sites had a tighter correlation (*r* = 0.65) compared to other traits with the exception of days to heading (*r* = 0.86). The average GY was 4.4 t ha^−1^at Anjiro and 4.2 t ha^−1^ at Ankazo and respective SWT means were 29.7 and 28.2 g plant^−1^ (Fig. 1). Neither trait differed significantly between sites.

Correlations between the two sites were low for GY (*r* = 0.15; *p* < 0.05) and slightly higher for SWT (*r* = 0.28; *p* < 0.001). However, at each site GY was positively correlated to SWT (*r* = 0.55 and *r* = 0.61 for Anjiro and Ankazo, respectively; *p* < 0.001 for both). Accessions showed large variation for HD, ranging from 60 to 127 days at Ankazo and from 64 to 129 days at Anjiro. The similar range and high correlation of *r* = 0.86 indicated that site effects were very small for HD. Late heading was associated with increased SWT at both sites but the effect of late heading on GY was site-specific, with a low but significantly positive effect at Anjiro (*r* = 0.32; *p* < 0.001) compared to a non-significant (negative) effect (*r* =  − 0.11; ns) in Ankazo (Fig. 1).

Interestingly, Zn concentrations showed weak correlations with other traits, except for a low and negative correlation with GY in Anjiro (*r* =  − 0.33; *p* < 0.001). Furthermore, the broad-sense heritability for Zn concentrations across sites was high (H^2^ = 0.79; Fig. S1), implying that genotype by site interaction effects for Zn concentrations were small. For that reason, the association mapping and GP were conducted with the across-site BLUE values to analyze the common genetic control across two sites.

**Fig. 2:**
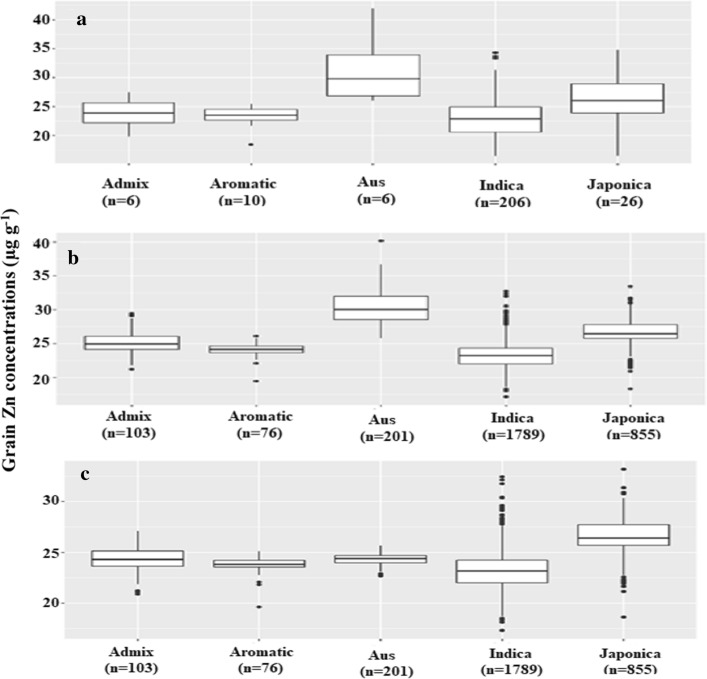
Variation in grain Zn concentrations in accessions from five rice sub-populations: **a** measured data of the training set (*n* = 253); **b** predicted values of the entire 3 K set using the full training set (*n* = 253); **c** predicted values of the entire 3 K set using a training set excluding six aus accessions (*n* = 247)

The 253 accessions tested belonged primarily to the *indica* sub-species of rice, the second biggest group were *japonica* accessions while other sub-populations were represented by only 6–10 individuals (Fig. 2a). The focus on mostly *indica* accessions was due to the preference for *indica*-type varieties by lowland rice growers and consumers in Madagascar. The *aus* sub-species group had the highest average grain Zn concentration (33.5 µg g^−1^), compared with an average of 25.1 µg g^−1^ for the *indica* group and 28.4 µg g^−1^ for the *japonica* group.

### Genome-wide associations for grain Zn concentrations

A multi-locus GWAS approach considering results from six multi-locus analysis methods was employed to identify genetic associations with grain Zn concentrations and associations were considered significant when the estimated LOD surpassed the threshold of 3.0 in at least three methods. Based on these criteria, eight loci associated with grain Zn concentrations were detected on chromosomes 2, 4, 8, 10, 11 and 12 (Table 1). The full list of all QTN with a LOD > 3 in any of the six methods is shown in Table S1 and the corresponding Manhattan plots in Fig. S2.

**Table 1:** Associations for grain Zn concentrations detected (with LOD > 3.0) in at least three of the six multi-locus approaches employed. For *R*^2^ and QTN effects, highest estimates by any of the significant approaches are shown

QTN	Chromosome	Position	mrMLM	FASTmrMLM	FASTmrEMMA	pkWmEB	pLARmEB	ISIS EM-BLASSO	*R*^2^	MAF (%)	QTN effect (µg Zn g^−1^)
2.1	2	18,697,369	5.1	3.2	4.9	3.2			5.5	45.9	1.2
4.1	4	20,025,747		7.8		3.7	4.2		5.9	20.6	1.2
8.1	8	26,505,039		7.5	6.5	6.7	5.6		3.0	10.7	1.9
10.1	10	14,217,374	4.4	3.6		7.6	4.1	3.9	3.5	5.5	1.5
11.1	11	26,546,816	4.3	6.2		8.6	6.2		4.9	13.0	1.2
11.2	11	27,604,708		6.0	3.2	7.4			3.2	15.6	− 1.4
11.3	11	28,757,650	15.2	10.4		13.6	16.4	3.6	15.4	4.4	3.7
12.1	12	7,184,806	3.1	4.8		3.9			2.1	9.7	0.9

The strongest peak identified in terms of maximum LOD value (16.4), number of approaches identifying the locus (5) and consistently high QTN effect estimates (1.69–3.69 µg Zn g^−1^) was QTN_11.3 at 28,757,650 bp on chromosome 11 (Table 1; Table S1). The minor allele frequency (MAF) at this locus was 4.4% and the *R*^2^ was 15.4. The remaining seven loci had comparatively minor effects with *R*^2^ estimates of 2.1–5.9 and maximum QTN effect estimates between 0.9 and 1.9 µg Zn g^−1^ (Table 1).

**Fig. 3:**
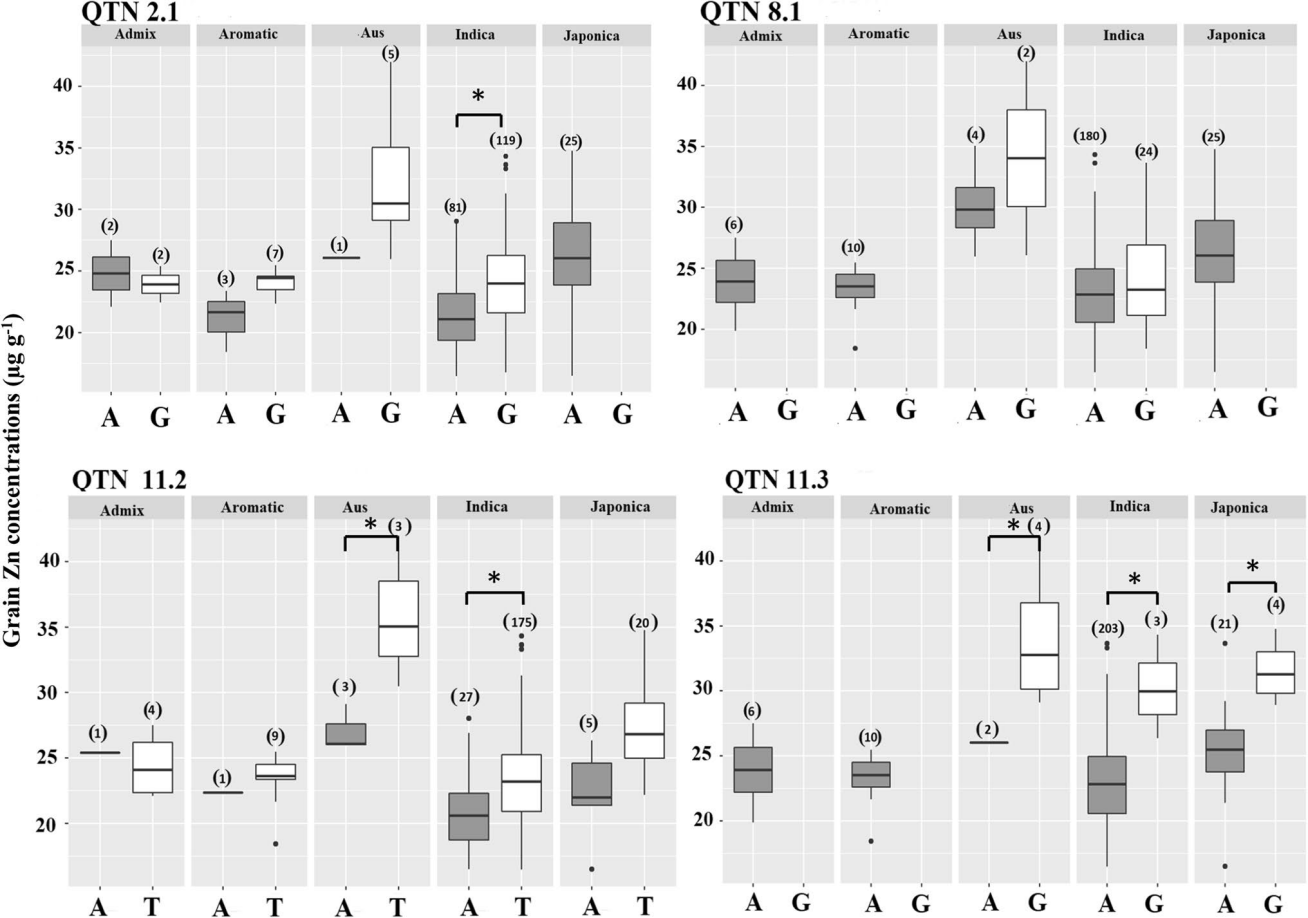
Grain Zn concentrations for the two allelic groups in the five sub-populations for QTN_2.1, QTN_8.1, QTN_11.2 and QTN_11.3. Numbers above bars indicate the number of accessions in the respective group

At each QTN we investigated to what extent the minor allele and the allele increasing grain Zn concentrations was associated with different rice sub-populations (Fig. 3). For QTN_2.1 allelic variation was detected in all sub-species except for *japonica* but differences were only significant in the *indica* group. QTN_8.1 had allelic variation within the *aus* and *indica* groups but mean differences were not significant. QTN_11.2 allelic variants existed within all sub-species and the minor allele significantly increased grain Zn in the *aus* and *indica* groups. For the strongest QTN_11.3, the minor allele was associated with higher grain Zn concentrations in the *aus*, *indica* and *japonica* sub-species (Fig. 3). At QTN on chromosomes 10 and 12 allelic variation was only detected within the *indica* group and the minor allele increased grain Zn (Fig. S3). In only one case (chromosome 11: 27,604,708), the minor allele was associated with reduced grain Zn and the allelic difference was only pronounced in the *aus* and *japonica* groups where it reduced grain Zn by 24 and 19%, respectively.

For the most influential locus QTN_11.3, we delimited the linkage block surrounding significant QTNs. Strong linkage that would define a clear block was not detected but similarities between SNP genotypes were suggestive of a linkage block extending from 28.681 to 28.798 Mbp (Fig. S4). This region contained 26 gene models of which 18 were functionally annotated (Table S2). One gene family was strongly overrepresented at this locus as 11 genes were annotated as glucosyl hydrolases or, more specifically, as either class III chitinase homologs or xylanase inhibitors. In addition, two “thaumatin family domain containing proteins” and two Zinc finger proteins were annotated in the target region.

### Genomic prediction for grain Zn concentrations

Utilizing the same GWAS dataset as a training set, a GP model was developed to predict grain Zn concentrations of the entire set of 3 K accessions. The full model including all 253 training accessions predicted grain Zn concentrations to range from 17.1 µg Zn g^−1^ to as high as 40.2 µg Zn g^−1^ (Fig. 2b). Differences between subpopulations were pronounced, with the *aus* group having highest predicted values and an average of 30.3 µg Zn g^−1^. The second highest average was predicted for the *japonica* group (26.7 µg Zn g^−1^) and the lowest for the *indica* group (23.2 µg Zn g^−1^). In fact, only members of the *aus* sub-species were found among the top 20 predicted accessions (Table S3) and IRIS_313_9368, which had the highest grain Zn concentrations in the training set (42.0 µg Zn g^−1^), was also the highest predicted accession (40.2 µg Zn g^−1^).

**Fig. 4:**
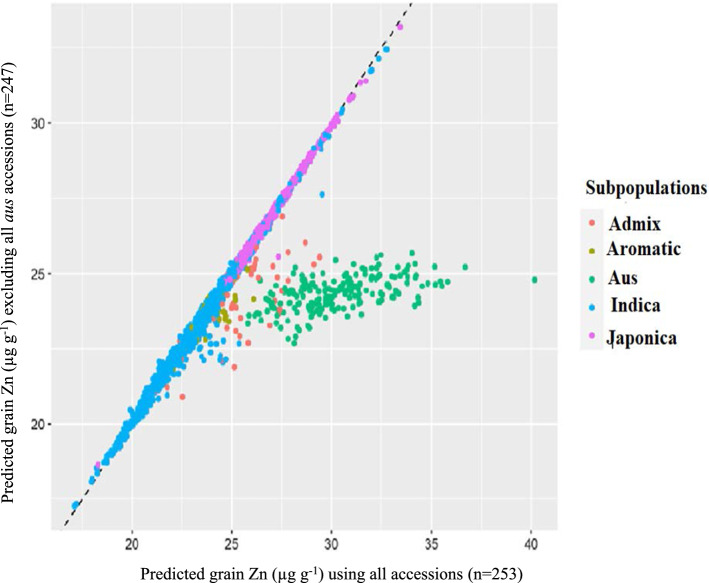
Predicted grain Zn concentrations of the entire 3 K set of accessions available at the IRRI gene bank based on two different training sets. Predictions shown on the x-axis are based on the entire set of 253 accessions tested in Madagascar, whereas predictions on the y-axis are based on only 247 accessions with all six members of the aus sub-population omitted

The training set contained only six *aus* accessions, among which IRIS_313_9368 may have been highly influential. To test to what extent the small number of *aus* accessions may have skewed predictions, two additional GP models were tested, a 2nd model excluding IRIS_313_9368 and a 3rd model excluding all six *aus*. Predicted values of the 3rd model are shown in comparison to the full model in Fig. 4. Excluding *aus* from the training set did not have major effects on predicted values for the four non-*aus* sub-populations, but strongly decreased the predicted grain Zn concentrations of the 201 *aus* accessions among the 3 K set. Their average decreased from 30.3 to only 24.3 µg Zn g^−1^, which was lower than the predicted average of the *japonica group* (26.6 µg Zn g^−1^) using the same training set (Fig. 2c). Only excluding IRIS_313_9368 did not have comparably strong effects, though the predicted Zn concentrations in *aus* accessions decreased from the full model (Fig. S5).

Tenfold cross-validation was performed to evaluate the accuracy of predictions for the full model and the one excluding *aus* accessions. When using all phenotyped accessions, average prediction accuracy of the ten replications was *r* = 0.51 with the standard deviation (SD) of 0.02. This dropped slightly to *r* = 0.49 (SD = 0.01) for the 2nd model and to *r* = 0.48 (SD = 0.01) for the 3rd model with *aus* excluded.

**Fig. 5:**
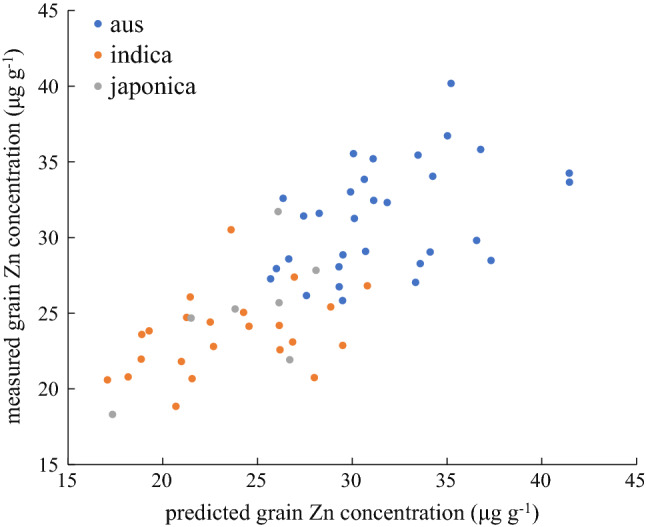
Independent validation of predicted grain Zn concentrations in a set of 61 accessions imported from the IRRI gene bank. Predicted values are based on the training set of 253 accessions grown in Madagascar and measured values are from seed distributed by the IRRI gene bank

For an additional and independent confirmation of prediction results, grain Zn concentrations were determined in a different subset of accessions selected from the 3 K set. These belonged to the *aus* (*n* = 30), *indica* (*n* = 24) and *japonica* (*n* = 7) subpopulations (Table S4). Seeds analyzed had been imported directly from the IRRI genebank and were thus not grown in Madagascar (though there was a small overlap with accessions in the field in Madagascar, *n* = 11). The correlation between measured and predicted values was *r* = 0.74 (Fig. 5) and correlations with the 2nd model (*r* = 0.66) or 3rd model (*r* = 0.35) were lower (data not shown). Predicted mean values separated the *aus* group (average 31.3 µg Zn g^−1^) from the *indica* (23.6 µg g^−1^) and *japonica* (25.1 µg g^−1^) groups and measured means were within 1.0 µg Zn g^−1^ of predicted group means (31.7, 23.4 and 24.2 µg Zn g^−1^, respectively). For the main locus identified on chromosome 11 in GWAS (QTN_11.3), the positive minor allele was present in 12 of the 61 accessions. The allelic effect appeared to be significant with an average Zn concentration of 32.4 µg Zn g^−1^ for the minor compared to 26.3 µg Zn g^−1^ for the major allele (Table S5); however, all accessions with the minor allele belonged to the *aus* group and within that sub-species, allelic groups did not differ (32.4 vs. 31.1 µg Zn g^−1^).

**Fig. 6:**
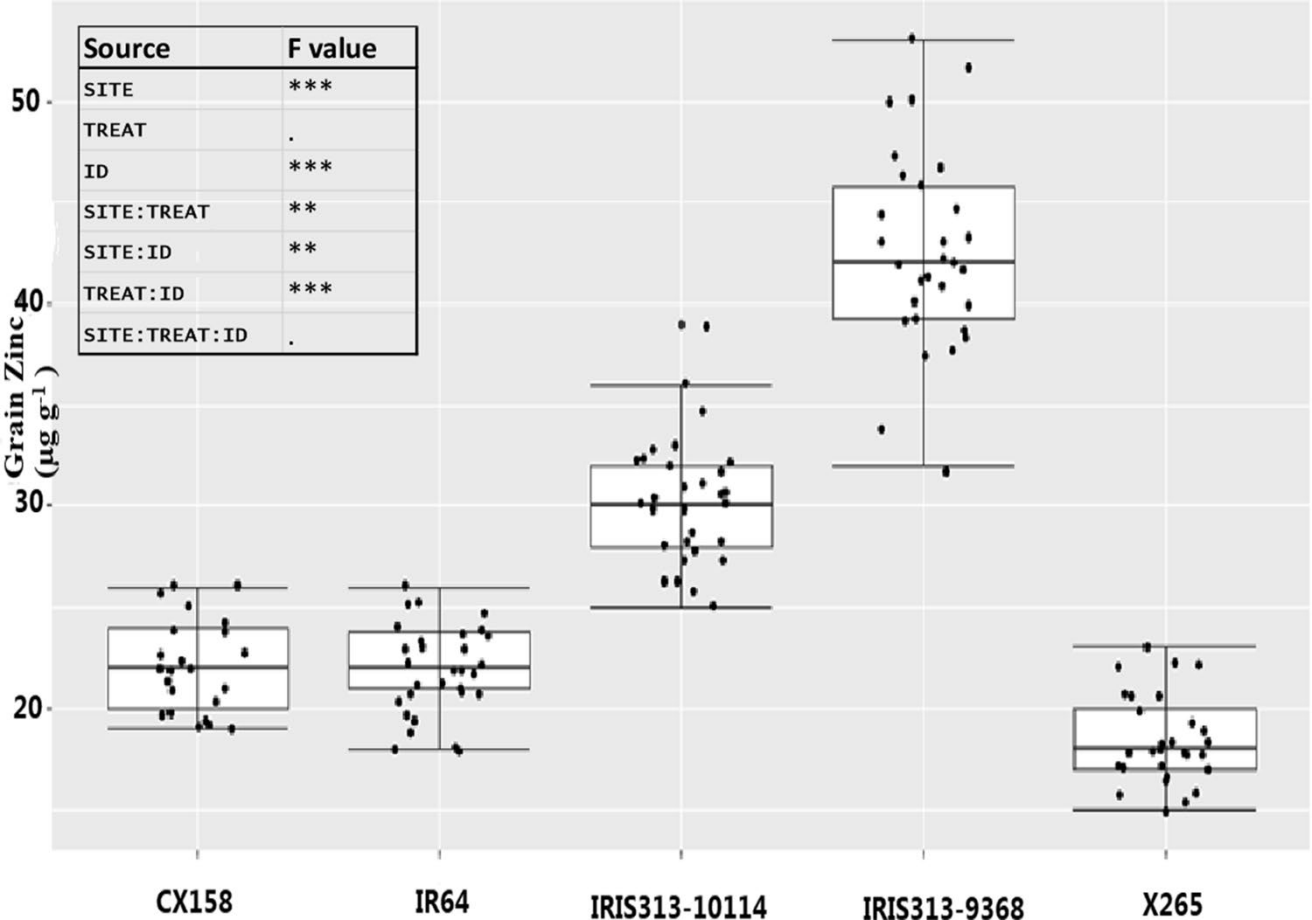
Variation in grain Zn concentration of five rice accessions across five field sites and two fertilizer treatments (no input and 120 kg ha^−1^ NPK compound fertilizer). Differences between accessions were the dominant source of variation, followed by differences between sites (see inlet of ANOVA table)

The second confirmatory experiment was conducted with potential high-Zn donors at five field sites and with two fertilizer treatments in Madagascar. The ANOVA indicated that genotypic differences were the dominant source of variation in this dataset (Fig. 6) and potential high-Zn donor IRIS313-9368 (*aus*) was consistently superior to other accessions, irrespective of sites and fertilizer treatments. With an average of 42.5 µg Zn g^−1^, it surpassed its predicted grain Zn concentration of 40.2 µg Zn g^−1^. Furthermore, it had almost twice the Zn concentration of IR64 (21.7 µg g^−1^) and more than twice compared to the Malagasy check X265 (18.6 µg g^−1^). Other tested accessions were the top predicted *indica* CX158 and IRIS313-10,114 and while CX158 failed to reach its predicted value of 32.0 µg Zn g^−1^, IRIS313-10,114 matched its predicted value of 30.5 µg g^−1^ to within 1% and was consistently superior to the check varieties, irrespective of site or fertilizer effects (Fig. 6). As in the training set, plants had higher average grain Zn concentrations at both field sites in Anjiro (28.6–29.6 µg Zn g^−1^) compared to Ankazo village (26.5 µg Zn g^−1^), with Behenji (25.0–25.9 µg Zn g^−1^) being lowest. Applying NPK fertilizer had a small positive effect, increasing average grain Zn concentrations from 26.6 to 27.7 µg Zn g^−1^) but this effect was only significant in two of the five sites (data not shown).

## Discussion

Experiments were conducted with a diverse set of gene bank accessions and more than twofold variation in grain Zn concentrations were detected. Other traits such as GY or HD also varied considerably, but correlations between this variation and grain Zn concentrations were low (Fig. 1). Large differences in GY could have affected grain Zn concentrations, due to a possible dilution of Zn in the greater grain biomass of higher-yielding accessions and of the reverse effect in very low-yielding accessions. While excluding high-yielding varieties was no option because such material should be the target of any breeding program, we had omitted accessions with extremely low yield at any of the two sites as we considered these to be not sufficiently adapted to local conditions to provide reliable data. Possibly as a result of this precaution the correlation between GY and grain Zn concentrations remained very weak (*r* = − 0.27; Fig. S1) and likely did not affect outcomes of the GWAS and GS studies. In addition, the heritability for grain Zn concentrations (H^2^ = 0.79) was larger than for GY (H^2^ = 0.40). High heritability (> 0.70) for grain Zn has also been reported elsewhere (Swamy et al. 2016, 2018; Baertschi et al. 2021).

A rather high heritability and good correlations for grain Zn concentrations between different sites did, however, not mean site effects were absent. Grain Zn concentrations differed significantly and consistently between sites, with samples from Anjiro village (25.4 µg g^−1^) having significantly higher grain Zn concentrations compared to the Ankazo site (21.6 µg g^−1^) and this may have been due to poor drainage in Ankazo, which could lower Zn availability due to a more reduced soil state. Nevertheless, neither site can be described as Zn deficient considering our observed ranges were comparable to or higher than in similar studies (Norton et al. 2014; Swamy et al. 2018; Rao et al. 2020).

All rice grain analyzed was unpolished brown rice, which was primarily due to the fact that high-quality milling equipment capable of polishing rice without contaminating samples with Zn during the milling process was not available in Madagascar. To what extent our analysis of brown rice could have affected results and conclusions were briefly assessed by polishing a small sub-sample of grain and results indicated that the average grain Zn concentrations decreased by 18% from 33.2 µg g^−1^ in brown rice to 27.2 µg g^−1^ in polished rice (Fig. S6), which is similar to reductions reported elsewhere (Suman et al. 2021). Interestingly, the reduction was larger in low-Zn accessions (− 24.5%) compared to high-Zn accessions (− 14.2%), which would indicate that using brown rice samples would not have induced a bias in favor of high-Zn accessions in our analyses.

### Differences between rice sub-populations

The association panel used was predominantly of the *indica* sub-population with smaller additions from the *aus*, *japonica*, *aromatic* and *admix* groups, and measured grain Zn concentrations indicated significantly higher concentrations in the *aus* group (Fig. 2). The average of the *aus* group was 35.6% higher compared to the *indica* group and this superiority could subsequently be substantiated (+ 35.4%) in the confirmatory set including a much larger proportion of *aus* accessions. Considering that *indica* are the predominant group of varieties grown by lowland rice farmers of Madagascar and many other biofortification target countries, *aus* accessions identified here experimentally or through GP should be considered as potential donors in biofortification programs. Norton et al. (2014) reached a similar conclusion as 3 of the 5 high-Zn donor accessions identified in that study belonged to the *aus* group.

The inclusion or omission of *aus* accessions did not affect the ability to predict grain Zn in non-*aus* groups but omitting the *aus* led to a strong under-estimation of grain Zn in that group (Figs. 2 and 4). Thus, some largely *aus*-specific genetic factors must exist that lead to the superior grain Zn in that group and a GP approach appears to accurately consider these. A similar conclusion about the need to include all sub-populations in the training set was reached by Grenier et al. (2015). Considering that many component traits lead to high grain Zn, it is likely that some of these component traits are only or at least predominantly expressed in the *aus* group. Such traits would make ideal breeding targets and physiological studies need to investigate if such *aus*-specific traits exist and which limiting step in the processes between Zn uptake, transport, retranslocation and endosperm loading they affect.

Understanding such bottleneck traits and the underlying genetic control may be key to increasing grain Zn concentrations in the predominantly *indica* modern cultivars. One possible aspect to study further in this regard is the tendency of *aus* accessions like IRIS313-9368 to rapidly senesce at maturity, whereas modern *indica* varieties have the tendency to remain comparatively green at maturity. More rapid senescence could favor Zn remobilization and translocation to grains.

Donors from the *aus* group have been used repeatedly to move alleles for tolerance to many abiotic stresses into the modern rice breeding pool (Heredia et al. 2021). It is furthermore interesting to note that several *aus* accessions possess high tolerance to Zn deficiency (Lee et al. 2018) due to their efficient Zn uptake capacity from highly reduced soil. While it is not known whether a link between this Zn uptake efficiency from Zn deficient soil and high grain Zn concentrations under normal Zn availability exists, it would be very interesting to pursue such a possibility further. The use of *aus* donors in rice breeding has typically involved the transfer of major genes or QTL through their marker-assisted introgression into modern breeding lines and to what extent this is a likely approach to improve grain Zn remains to be resolved.

### GWA and GP analysis

Results of Norton et al. (2014) and Swamy et al. (2018) suggest that grain Zn is a polygenic trait controlled by multiple small to medium effect loci. Employing a multi-locus GWAS approach should therefore be more suitable in identifying loci controlling grain Zn concentrations compared to single-locus models that test one locus at a time without considering interactions between loci (Xu et al. 2018). A weakness of the single-locus GWAS analysis is the problem of false positives and negatives and this is better balanced in the multi-locus association analysis employed here, which eliminates the need for a Bonferroni correction in multi-locus GWAS (Wang et al. 2016).

This study identified eight QTN of which seven had minor effects while the QTN on chromosome 11 (28,757,650) can be considered a medium-effect locus. It is attributed to a rare allele present in accessions of the *aus*, *japonica* and *indica* sub-species and the difference in average grain Zn concentration between the minor (32.1 µg Zn g^−1^) and major (23.2 µg Zn g^−1^) allele groups at this QTN is 8.9 µg Zn g^−1^ (+ 38%). This contrasts with the estimated QTN effect of 3.69 µg Zn g^−1^ obtained by the multi-locus analysis. This would indicate a strong over-estimation of QTN effects if individual loci are investigated in isolation and that the multi-locus model may provide lower but more realistic estimates of QTN effects.

Other QTL or GWA studies have identified loci on chromosome 11 (summarized by Swamy et al. 2016, 2018) but these do not overlap with QTN_11.3 identified here. Conversely, we did not detect an otherwise commonly identified locus on chromosome 7 that is potentially linked to the *OsNAS3* gene considered a prime candidate for increasing grain mineral concentration (Johnson et al. 2011). Predicted gene models for the candidate region at QTN_11.3 (28.681–28.798 Mbp) did not contain genes previously associated with Zn metabolism or transport. Instead, a cluster of 11 genes belonging to the glucosyl hydrolase family (class III chitinase homologs or xylanase inhibitors) were present. However, it is beyond the scope of this paper to further analyze any potential role of these genes.

Whereas several QTL and GWAS studies have been reported in the literature this is only the second report applying GP for grain Zn concentrations in rice. In contrast to the work by Baertschi et al. (2021) that focused on assessing the potential of GP models for early selection of families to improve upland rice synthetic populations, the aim of the GP approach taken here is to predict grain Zn concentrations of gene bank accessions in lowland rice. If successful, this would allow for a very efficient search of potential new donors for high grain Zn concentrations. The prediction accuracy of 0.51 achieved in this study is similar to the PA ranging from 0.33 to 0.69 in wheat (Velu et al. 2016), of 0.43–0.73 reported for maize (Mageto et al. 2020) and of 0.51 in upland rice (Baertschi et al. 2021). The present study was conducted in two low-input farmers’ fields rather than under the more controlled conditions one may encounter on research farms and that the PA achieved here is comparable to PAs reported from research farms is further suggestive of GP being a suitable approach for identifying potential donors from gene banks. It is furthermore noteworthy that the GP model based on field data from Madagascar was able to reliably predict (*r* = 0.74) grain Zn concentrations of the confirmation set that had been grown on the IRRI farm in the Philippines, which represents a more favorable environment compared to the low-input farmers’ fields providing the data for the training set. Thus, the confirmation outside the training environment lends further credibility to the predictive ability of GP for grain Zn concentrations.

Such robustness across environments would offer options to further economize resources through sparse testing of only part of the entire training set at each site or environment. Baertschi et al. (2021) suggested optimizing the GP scheme by evaluating small training sets and using phenotypic correlation between sites to calibrate the model, and in their case the phenotypic correlation for grain zinc concentration between sites (*r*^2^ > 0.41) was similar to what was achieved here.

In a review of genomic approaches to biofortification of cereals, Koç and Karayigit (2021) concluded that conventional breeding would be the most sustainable, low cost and easily adoptable strategy. Our results concur inasmuch none of the QTN identified would be influential enough to be rapidly employed in marker-assisted selection. However, the success of GP in predicting grain Zn concentrations, here of gene bank accessions, but elsewhere in a rice breeding population (Baertschi et al. 2021), may offer opportunities, especially where genomic selection of other traits is already practiced. It should furthermore facilitate utilizing the high-Zn donors identified here, as breeders may be reluctant to employ such exotic material in a conventional elite breeding program. As efforts are under way to mainstream biofortification traits in crop breeding (Virk et al. 2021), it seems worthwhile to include grain Zn concentrations as one of the traits targeted in genomic selection.

## Conclusions

Data obtained from field experiments conducted in Madagascar enabled us to successfully predict grain Zn concentrations among a set of gene bank accessions, thereby identifying potential donors for use in Zn biofortification breeding. The most promising donors all belonged to the *aus* sub-species of rice and to significantly increase Zn concentrations in the lowland rice breeding pool, which is predominantly belonging to the *indica* sub-species, it appears necessary to rely on *aus* introgressions. Donor of the *aus* group has been used repeatedly for the introgression of major abiotic stress tolerance loci through their marker-assisted introgression. This approach is less likely to be successful for the improvement of grain Zn concentrations as none of the identified loci identified here or elsewhere appear strong enough to raise grain Zn concentrations by the targeted 50% or more. Being a polygenic trait, the improvement of grain Zn concentrations would likely require the transfer of many small-effect loci simultaneously. Since we have shown the suitability of GP in identifying high-Zn donors, it can be expected that breeding populations developed from such donors could achieve target grain Zn concentrations if a similar genomic selection approach was used during the variety development process.

## Supplementary Information

Below is the link to the electronic supplementary material.

Supplementary file1 (PPTX 154 kb)

Supplementary file2 (PPTX 2951 kb)

Supplementary file3 (PPTX 253 kb)

Supplementary file4 (PPTX 1333 kb)

Supplementary file5 (PPTX 94 kb)

Supplementary file6 (PPTX 294 kb)

Supplementary file7 (XLSX 18 kb)

Supplementary file8 (XLSX 12 kb)

Supplementary file9 (DOCX 16 kb)

Supplementary file10 (XLSX 16 kb)

Supplementary file11 (XLSX 11 kb)

## Data Availability

Genotypic data used in the study are publicly available at https://snp-seek.irri.org/_snp.zul.
