# Supplementary material for: Genomic prediction of zinc-biofortification potential in rice gene bank accessions
Source: Theor Appl Genet. 2022 May 26;135(7):2265–78. doi: 10.1007/s00122-022-04110-2 (PMC9271118; doi:10.1007/s00122-022-04110-2)
Supplement: Supplementary file 1 — Supplementary file1 (PPTX 154 kb) [file 122_2022_4110_MOESM1_ESM.pptx]

## Slide 1
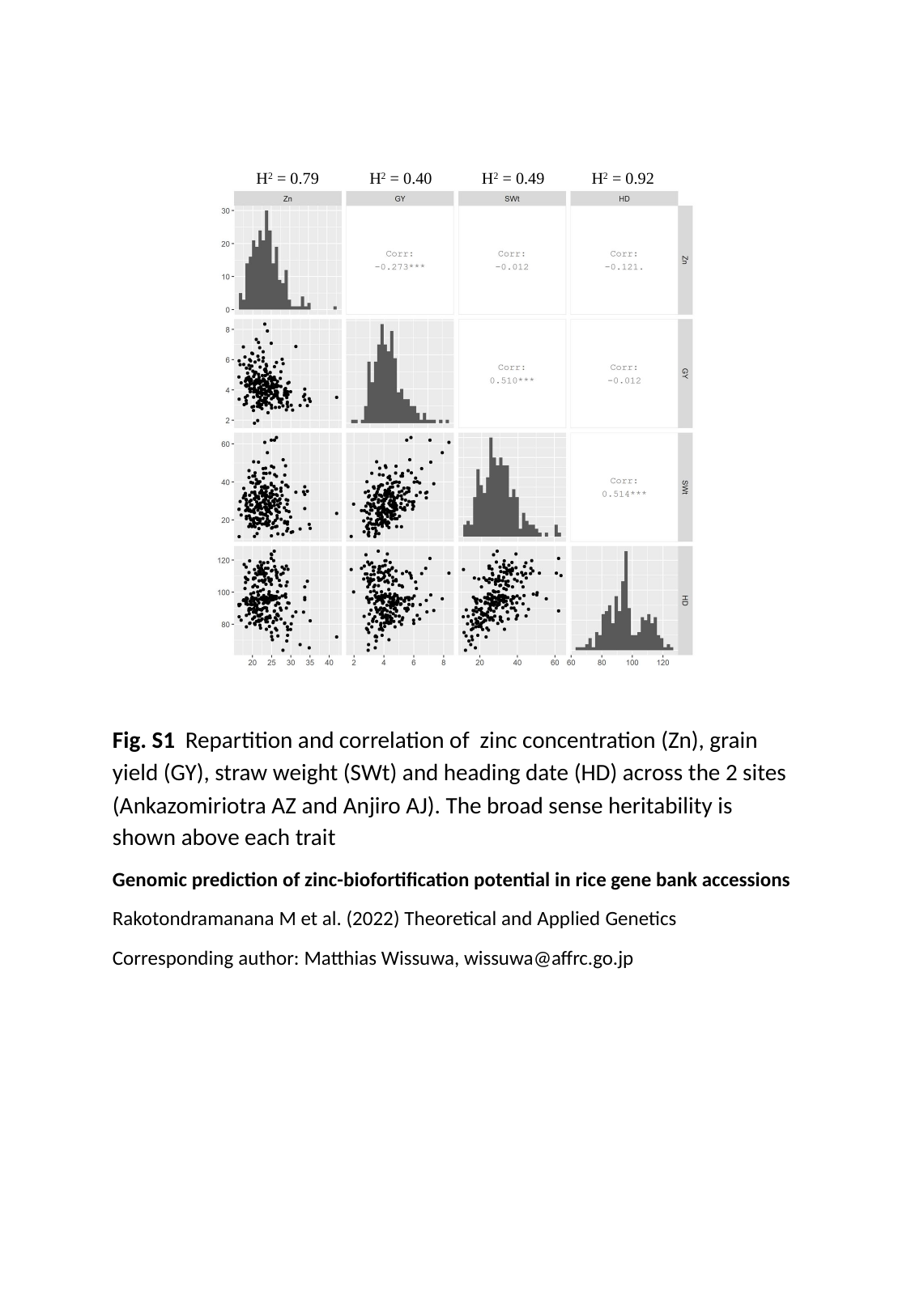

H2 = 0.40
H2 = 0.49
H2 = 0.92
H2 = 0.79
Fig. S1  Repartition and correlation of zinc concentration (Zn), grain yield (GY), straw weight (SWt) and heading date (HD) across the 2 sites (Ankazomiriotra AZ and Anjiro AJ). The broad sense heritability is shown above each trait
Genomic prediction of zinc-biofortification potential in rice gene bank accessions
Rakotondramanana M et al. (2022) Theoretical and Applied Genetics
Corresponding author: Matthias Wissuwa, wissuwa@affrc.go.jp
