# Supplementary material for: Genomic prediction of zinc-biofortification potential in rice gene bank accessions
Source: Theor Appl Genet. 2022 May 26;135(7):2265–78. doi: 10.1007/s00122-022-04110-2 (PMC9271118; doi:10.1007/s00122-022-04110-2)
Supplement: Supplementary file 2 — Supplementary file2 (PPTX 2951 kb) [file 122_2022_4110_MOESM2_ESM.pptx]

## Slide 1
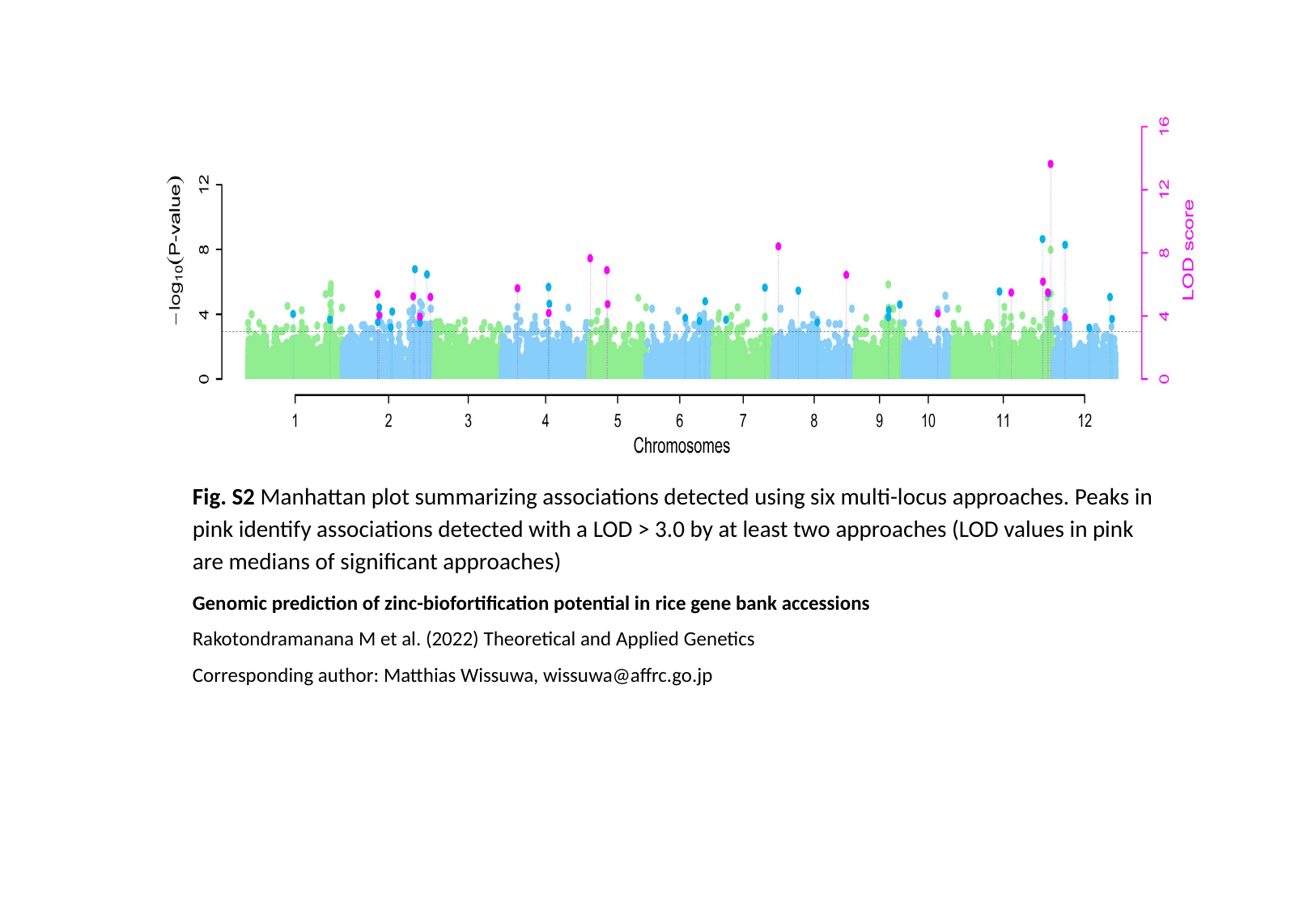

Fig. S2 Manhattan plot summarizing associations detected using six multi-locus approaches. Peaks in pink identify associations detected with a LOD > 3.0 by at least two approaches (LOD values in pink are medians of significant approaches)
Genomic prediction of zinc-biofortification potential in rice gene bank accessions
Rakotondramanana M et al. (2022) Theoretical and Applied Genetics
Corresponding author: Matthias Wissuwa, wissuwa@affrc.go.jp
