# Supplementary material for: Genomic prediction of zinc-biofortification potential in rice gene bank accessions
Source: Theor Appl Genet. 2022 May 26;135(7):2265–78. doi: 10.1007/s00122-022-04110-2 (PMC9271118; doi:10.1007/s00122-022-04110-2)
Supplement: Supplementary file 3 — Supplementary file3 (PPTX 253 kb) [file 122_2022_4110_MOESM3_ESM.pptx]

## Slide 1
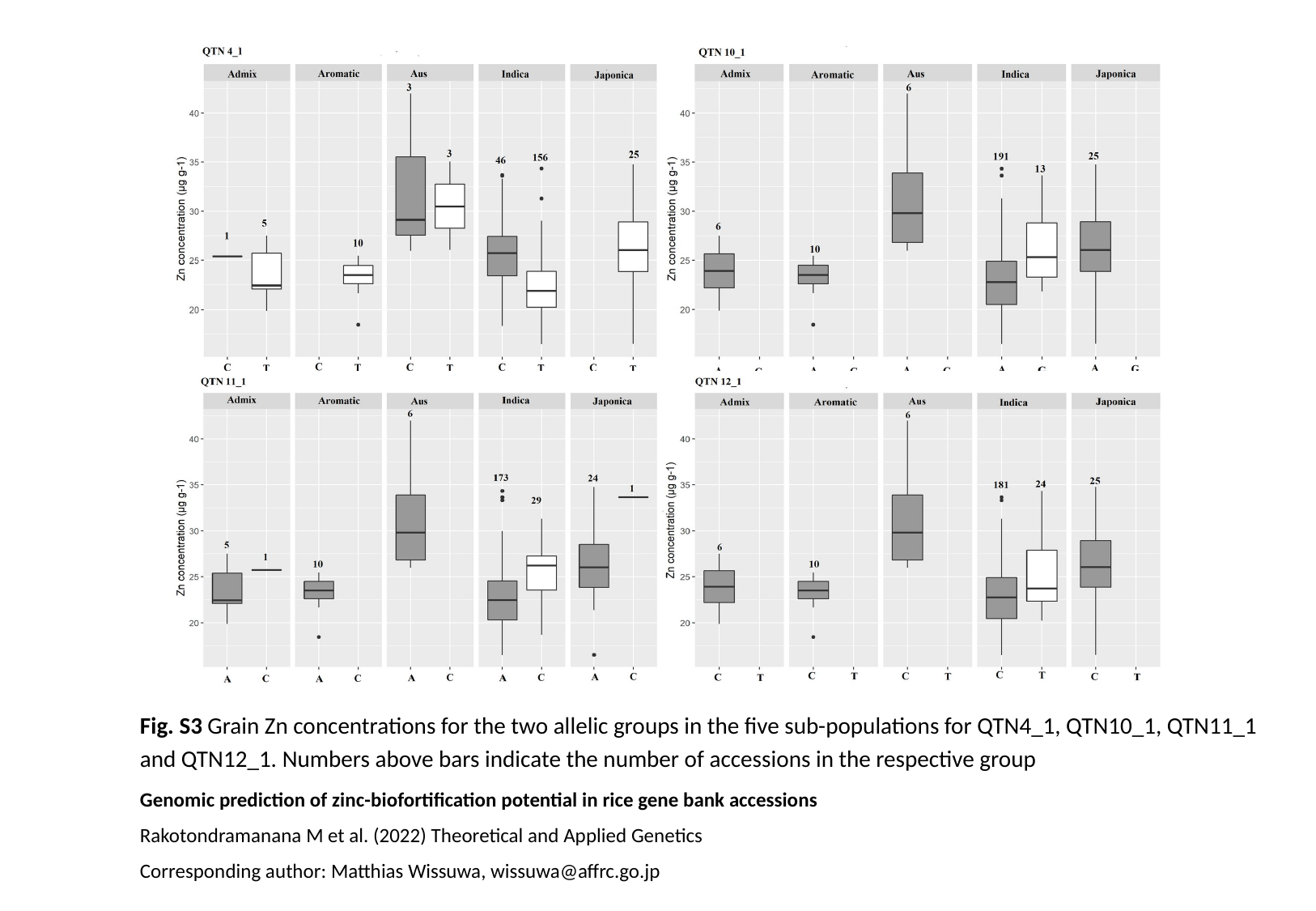

Fig. S3 Grain Zn concentrations for the two allelic groups in the five sub-populations for QTN4_1, QTN10_1, QTN11_1 and QTN12_1. Numbers above bars indicate the number of accessions in the respective group
Genomic prediction of zinc-biofortification potential in rice gene bank accessions
Rakotondramanana M et al. (2022) Theoretical and Applied Genetics
Corresponding author: Matthias Wissuwa, wissuwa@affrc.go.jp
