# Supplementary material for: Genomic prediction of zinc-biofortification potential in rice gene bank accessions
Source: Theor Appl Genet. 2022 May 26;135(7):2265–78. doi: 10.1007/s00122-022-04110-2 (PMC9271118; doi:10.1007/s00122-022-04110-2)
Supplement: Supplementary file 4 — Supplementary file4 (PPTX 1333 kb) [file 122_2022_4110_MOESM4_ESM.pptx]

## Slide 1
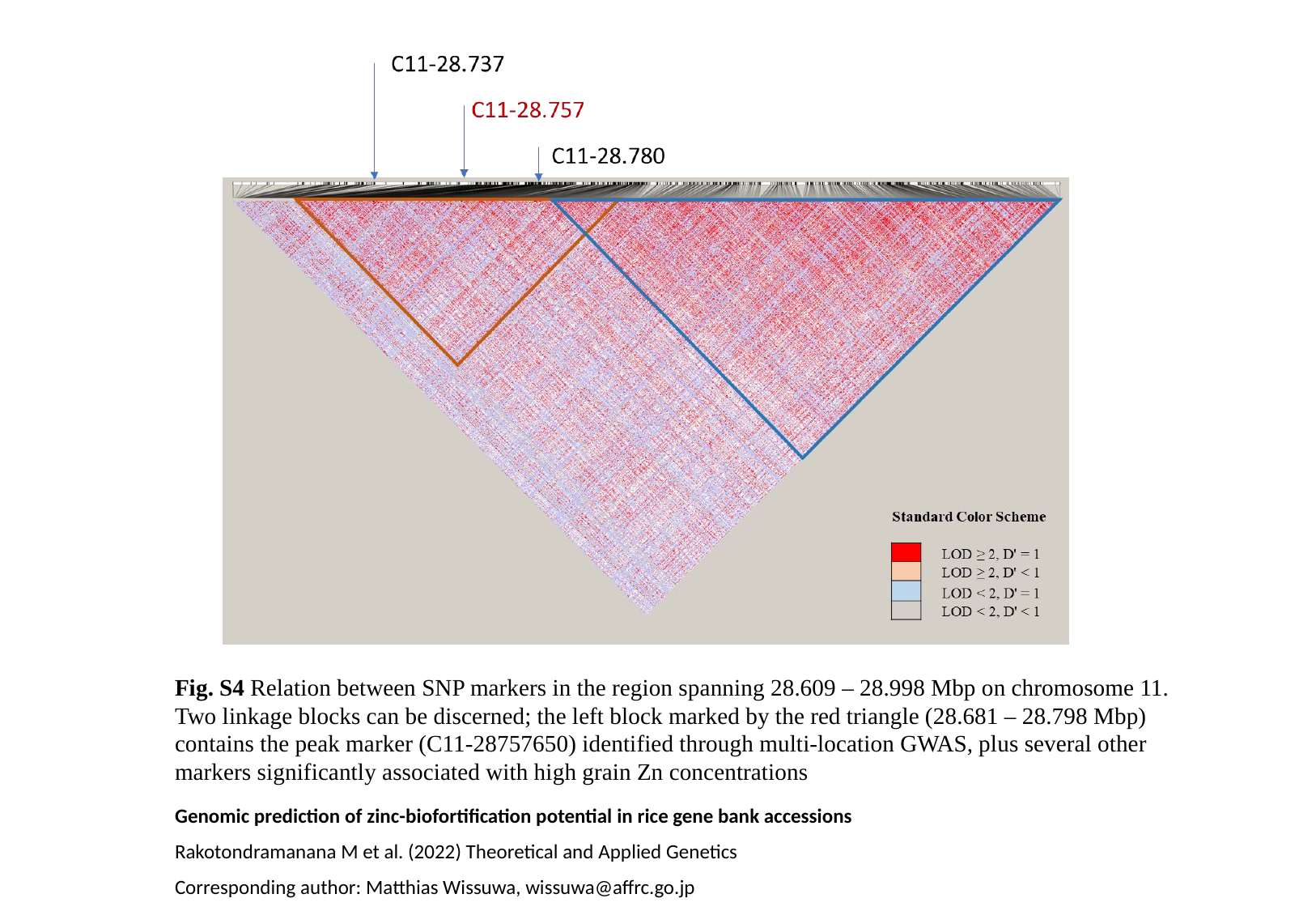

Fig. S4 Relation between SNP markers in the region spanning 28.609 – 28.998 Mbp on chromosome 11. Two linkage blocks can be discerned; the left block marked by the red triangle (28.681 – 28.798 Mbp) contains the peak marker (C11-28757650) identified through multi-location GWAS, plus several other markers significantly associated with high grain Zn concentrations
Genomic prediction of zinc-biofortification potential in rice gene bank accessions
Rakotondramanana M et al. (2022) Theoretical and Applied Genetics
Corresponding author: Matthias Wissuwa, wissuwa@affrc.go.jp
