# Supplementary material for: Genomic prediction of zinc-biofortification potential in rice gene bank accessions
Source: Theor Appl Genet. 2022 May 26;135(7):2265–78. doi: 10.1007/s00122-022-04110-2 (PMC9271118; doi:10.1007/s00122-022-04110-2)
Supplement: Supplementary file 5 — Supplementary file5 (PPTX 94 kb) [file 122_2022_4110_MOESM5_ESM.pptx]

## Slide 1
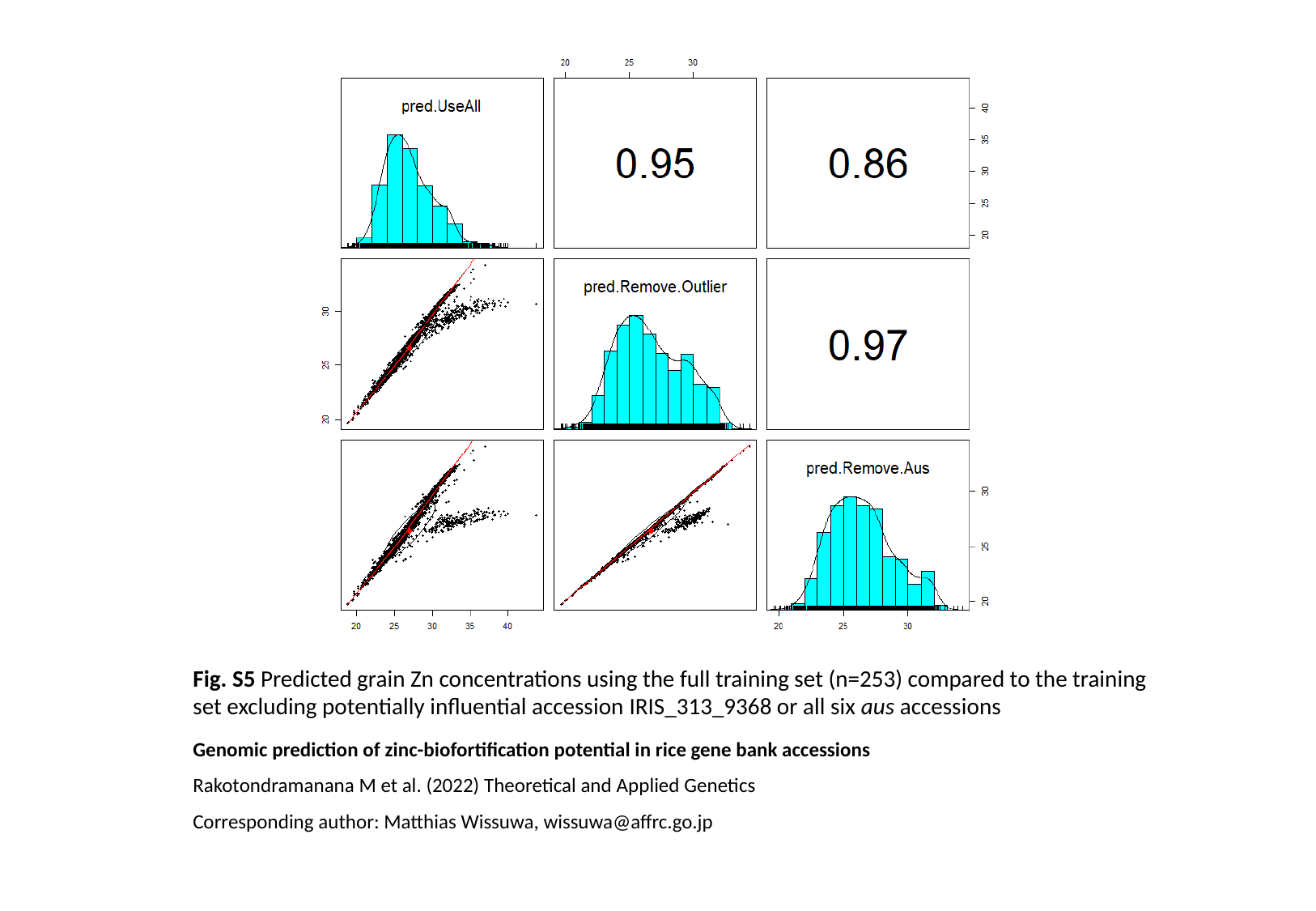

Fig. S5 Predicted grain Zn concentrations using the full training set (n=253) compared to the training set excluding potentially influential accession IRIS_313_9368 or all six aus accessions
Genomic prediction of zinc-biofortification potential in rice gene bank accessions
Rakotondramanana M et al. (2022) Theoretical and Applied Genetics
Corresponding author: Matthias Wissuwa, wissuwa@affrc.go.jp
