# Supplementary material for: Genomic prediction of zinc-biofortification potential in rice gene bank accessions
Source: Theor Appl Genet. 2022 May 26;135(7):2265–78. doi: 10.1007/s00122-022-04110-2 (PMC9271118; doi:10.1007/s00122-022-04110-2)
Supplement: Supplementary file 6 — Supplementary file6 (PPTX 294 kb) [file 122_2022_4110_MOESM6_ESM.pptx]

## Slide 1
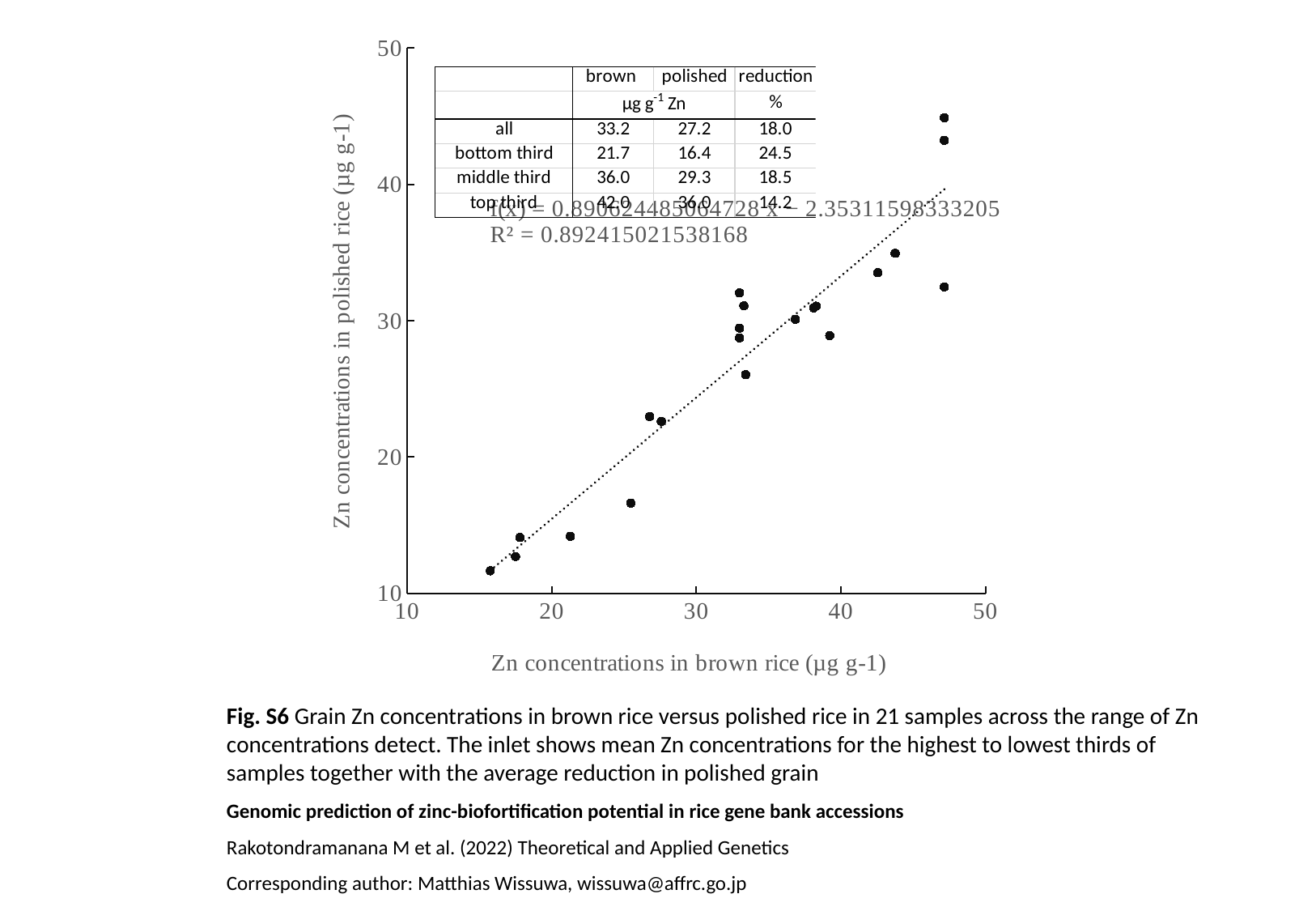

### Chart
| Category | |
|---|---|
Fig. S6 Grain Zn concentrations in brown rice versus polished rice in 21 samples across the range of Zn concentrations detect. The inlet shows mean Zn concentrations for the highest to lowest thirds of samples together with the average reduction in polished grain
Genomic prediction of zinc-biofortification potential in rice gene bank accessions
Rakotondramanana M et al. (2022) Theoretical and Applied Genetics
Corresponding author: Matthias Wissuwa, wissuwa@affrc.go.jp
