# Supplementary material for: Genomic prediction of zinc-biofortification potential in rice gene bank accessions
Source: Theor Appl Genet. 2022 May 26;135(7):2265–78. doi: 10.1007/s00122-022-04110-2 (PMC9271118; doi:10.1007/s00122-022-04110-2)
Supplement: Supplementary file 9 — Supplementary file9 (DOCX 16 kb) [file 122_2022_4110_MOESM9_ESM.docx]

**Table S3** Top 20 accessions among the 3K set with highest predicted grain Zn concentration using the full model. Values in brackets represent the standard error of the prediction.

| **IRIS.ID** | **predicted_Zn_Concentration** | **NAME** | **SUBPOPULATION** | **COUNTRY** |
| --- | --- | --- | --- | --- |
| IRIS_313_9368 | 40.2 (1.24) | CHANDARHAT::IRGC 25845-1 | aus | Bangladesh |
| IRIS_313_11174 | 36.7 (2.70) | SLO 19::IRGC 35157-1 | aus | India |
| IRIS_313_10594 | 35.8 (2.24) | DJ 29::IRGC 8505-1 | aus | Bangladesh |
| IRIS_313_10600 | 35.5 (2.71) | UCP 41::IRGC 8742-1 | aus | Bangladesh |
| IRIS_313_11048 | 35.4 (2.86) | AUS 171::IRGC 29004-1 | aus | Bangladesh |
| IRIS_313_11060 | 35.2 (2.78) | AUS 359::IRGC 29146-2 | aus | Bangladesh |
| IRIS_313_10604 | 35.2 (3.02) | DV 2::IRGC 8806-1 | aus | Bangladesh |
| IRIS_313_11322 | 34.8 (3.04) | JASURE AUS::IRGC 43860-1 | aus | Bangladesh |
| IRIS_313_10606 | 34.5 (3.01) | DV 110::IRGC 8855-1 | aus | Bangladesh |
| IRIS_313_10965 | 34.4 (2.90) | TILOKCHAN (SONABETHI)::IRGC 25927-1 | aus | Bangladesh |
| IRIS_313_11053 | 34.3 (2.99) | AUS 282::IRGC 29072-1 | aus | Bangladesh |
| IRIS_313_10587 | 34.2 (3.11) | DB 3::IRGC 8361-1 | aus | Bangladesh |
| IRIS_313_11324 | 34.2 (3.17) | KALABOKRI::IRGC 43872-1 | aus | Bangladesh |
| IRIS_313_11017 | 34.2 (3.21) | LOROI::IRGC 27567-1 | aus | Bangladesh |
| IRIS_313_12183 | 34.2 (1.25) | SETYA::IRGC 88654-1 | aus | Nepal |
| IRIS_313_10598 | 34.0 (3.24) | DD 126::IRGC 8667-1 | aus | Bangladesh |
| IRIS_313_8410 | 33.8 (3.18) | JABOR SAIL::IRGC 66831-1 | aus | Bangladesh |
| IRIS_313_11213 | 33.8 (3.24) | NARIKEL BADI::IRGC 37550-1 | aus | Bangladesh |
| IRIS_313_10595 | 33.8 (3.24) | DL 5::IRGC 8593-1 | aus | Bangladesh |
| IRIS_313_8073 | 33.8 (3.20) | DAWN CI 9534::GERVEX 481-C1 | aus |  |

**Genomic prediction of zinc-biofortification potential in rice gene bank accessions**

Rakotondramanana M et al. (2022) Theoretical and Applied Genetics.

Corresponding author: Matthias Wissuwa, wissuwa@affrc.go.jp
